# Oncogenic miRNA-182-5p Targets Smad4 and RECK in Human Bladder Cancer

**DOI:** 10.1371/journal.pone.0051056

**Published:** 2012-11-30

**Authors:** Hiroshi Hirata, Koji Ueno, Varahram Shahryari, Yuichiro Tanaka, Z. Laura Tabatabai, Yuji Hinoda, Rajvir Dahiya

**Affiliations:** 1 Department of Urology, San Francisco Veterans Affairs Medical Center and University of California San Francisco, San Francisco, California; 2 Department of Pathology, San Francisco Veterans Affairs Medical Center and University of California San Francisco, San Francisco, California; 3 Department of Oncology and Laboratory Medicine, Yamaguchi University Graduate School of Medicine, Yamaguchi, Japan; Wayne State University School of Medicine, United States of America

## Abstract

Onco-miR-182-5p has been reported to be over-expressed in bladder cancer (BC) tissues however a detailed functional analysis of miR-182-5p has not been carried out in BC. Therefore the purpose of this study was to: 1. conduct a functional analysis of miR-182-5p in bladder cancer, 2. assess its usefulness as a tumor marker, 3. identify miR-182-5p target genes in BC. Initially we found that miR-182-5p expression was significantly higher in bladder cancer compared to normal tissues and high miR-182-5p expression was associated with shorter overall survival in BC patients. To study the functional significance of miR-182-5p, we over-expressed miR-182-5p with miR-182-5p precursor and observed that cell proliferation, migration and invasion abilities were increased in BC cells. However cell apoptosis was inhibited by miR-182-5p. We also identified *Smad4* and *RECK* as potential target genes of miR-182-5p using several algorithms. 3′UTR luciferase activity of these target genes was significantly decreased and protein expression of these target genes was significantly up-regulated in miR-182-5p inhibitor transfected bladder cancer cells. MiR-182-5p also increased nuclear beta-catenin expression and while Smad4 repressed nuclear beta-catenin expression. In conclusion, our data suggests that miR-182-5p plays an important role as an oncogene by knocking down RECK and Smad4, resulting in activation of the Wnt-beta-catenin signaling pathway in bladder cancer.

## Introduction

Bladder cancer (BC) is the third leading cause of death among urological tumors and the most common histological type of bladder cancer is urothelial carcinoma (UC), formerly known as transitional cell carcinoma (TCC) [1]. Approximately 75% of patients are “non-muscle invasive UC (pTa, pTis, pT1) and have a 5-year survival rate of between 88–98% [2]. The common treatment for these patients is endoscopic resection [1], [3]. Patients with muscle invasive UC are usually treated with radical cystectomy or chemo radiotherapy [1], [4]. However half of muscle invasive UC patients develop subsequent metastatic disease after the first aggressive treatment [1], [5]. Previous studies have identified several potential molecular biomarkers for bladder cancer [6], [7]. Inactivation of tumor suppressor genes *TP53* and *Rb* and *Ras* oncogene activation have been regarded as important key players in bladder cancer carcinogenesis [6].

Activation of Wnt-beta-catenin signaling has also been studied and reported to be associated with cancer progression and poor prognosis in bladder cancer [8], [9]. Transforming growth factor beta (TGF-beta) plays a crucial role in embryonic development and pathogenesis of several diseases and cancer [10]. Evidence of crosstalk between TGF-beta and other signaling pathways including Wnt signaling have been reported [10]. Smad4 is a central intracellular signal transduction component of TGF-beta and recent studies have shown that Smad4 cooperates with beta-catenin in several caners [11]–[14].

RECK is crucial repressor of matrix metalloproteinases (MMPs) and previous studies have shown that RECK expression is significantly lower in bladder cancer tissues compared to normal urothelial tissues [15]–[17].

So far many microRNAs have been identified and reported to be important in several cancers [18]. MicroRNAs (miRNAs) are small non-coding RNAs, approximately 22 nucleotides in length, that are capable of regulating gene expression at both the transcription and translation levels [19]. MiRNAs bind to the 3′UTR of target mRNA and repress translation from mRNA to protein or induce mRNA cleavage and thereby regulate the expression of target genes [20].

In this study, we found that miR-182-5p was significantly higher in bladder cancer tissues compared to normal urothelial tissues and high miR-182-5p expression was significantly associated with shorter overall survival. So far there have been no reports about the function of miR-182-5p in bladder cancer. Thus we focused on miR-182-5p, performed functional analyses, identified several target genes of miR-182-5p using several algorithms and identified *Smad4* and *RECK* as target genes. Finally, we over-expressed these target genes (*Smad4* and *RECK*) in bladder cancer cells to examine the mechanism of miR-182-5p function.

## Results

### miRNA-182-5p Expression is Significantly Higher in Bladder Cancer Tissues and Associated with Shorter Overall Survival

**Figure 1:**
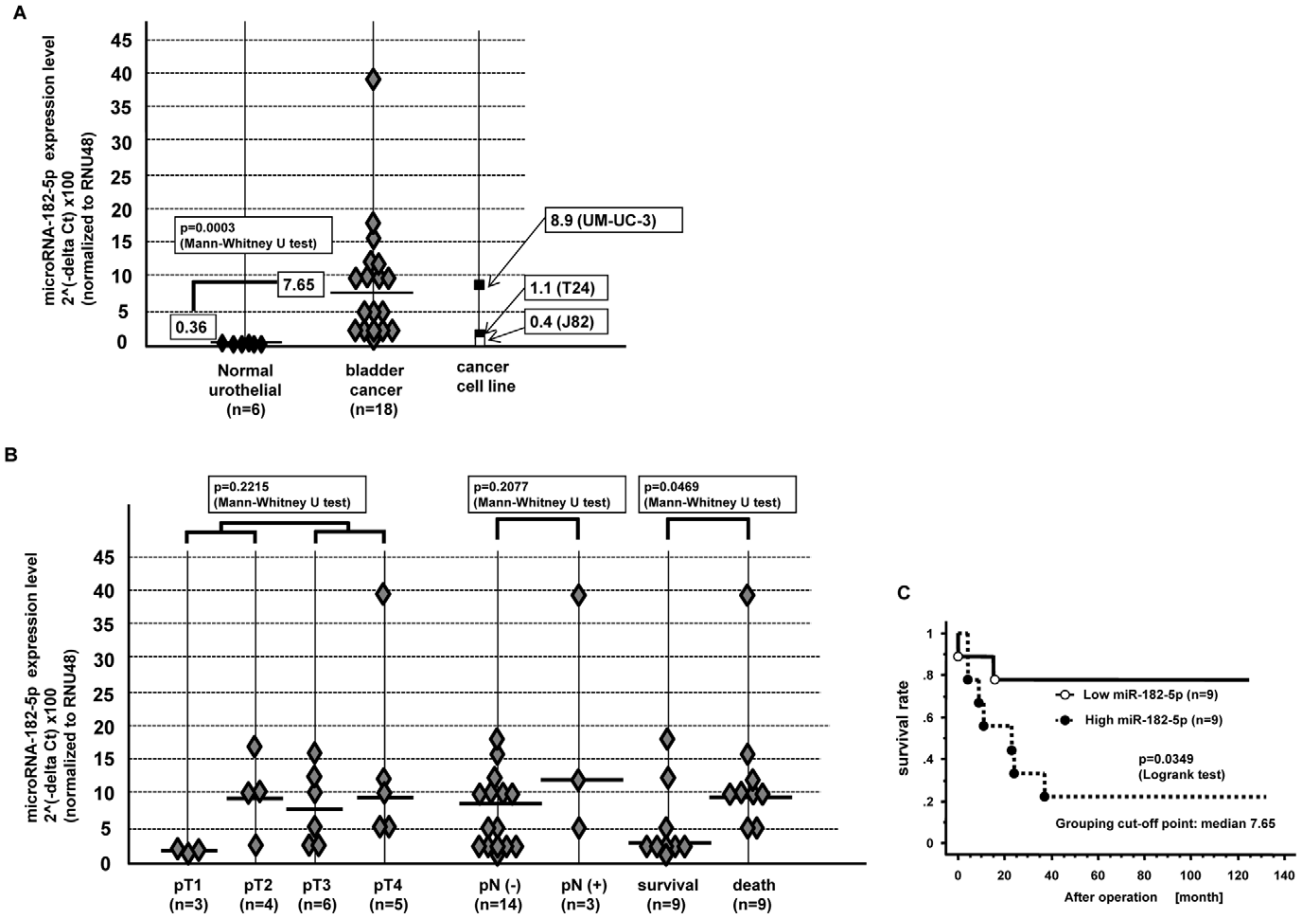
miR-182-5p expression and association with clinical parameters in bladder cancer tissues. A. miR-182-5p expression in clinical samples and bladder cancer cell lines, B. Association of miR-182-5p with clinic-pathological parameters, C. Kaplan Meier plots of overall survival.

We compared miRNA-182-5p expression levels in bladder cancer tissues (n = 18) and normal urothelial tissues (n = 6) by real-time PCR. The miR-182-5p expression was significantly higher in bladder cancer tissues (**Fig 1A**). We investigated the association of miR-182-5p and several clinical parameters as shown in **Figure 1** **-B** and observed that high miR-182-5p expression was significantly associated with shorter overall survival.

Regarding several other clinical parameters, no significant relationship was observed. We divided the 18 bladder cancer patients into two categories based on the median value and Kaplan Meier plots showed that overall survival was shorter in the high miR-182-5p expressing group (p value = 0.0349, Log-rank test) **(** **Figure 1** **-C)**.

### miRNA-182-5p Expression is Significantly Increased in Bladder Cancer Cell Lines

We compared miR-182-5p expression in several bladder cancer cell lines and its expression in T24 and UM-UC-3 cells was in the range of that in bladder cancer tissues. Thus we used these two bladder cancer cell lines for further experiments in this study (**Figure 1A**).

### Effect of microRNA-182-5p Over-expression on Cell Viability and Migration in Bladder Cancer Cell Lines

**Figure 2:**
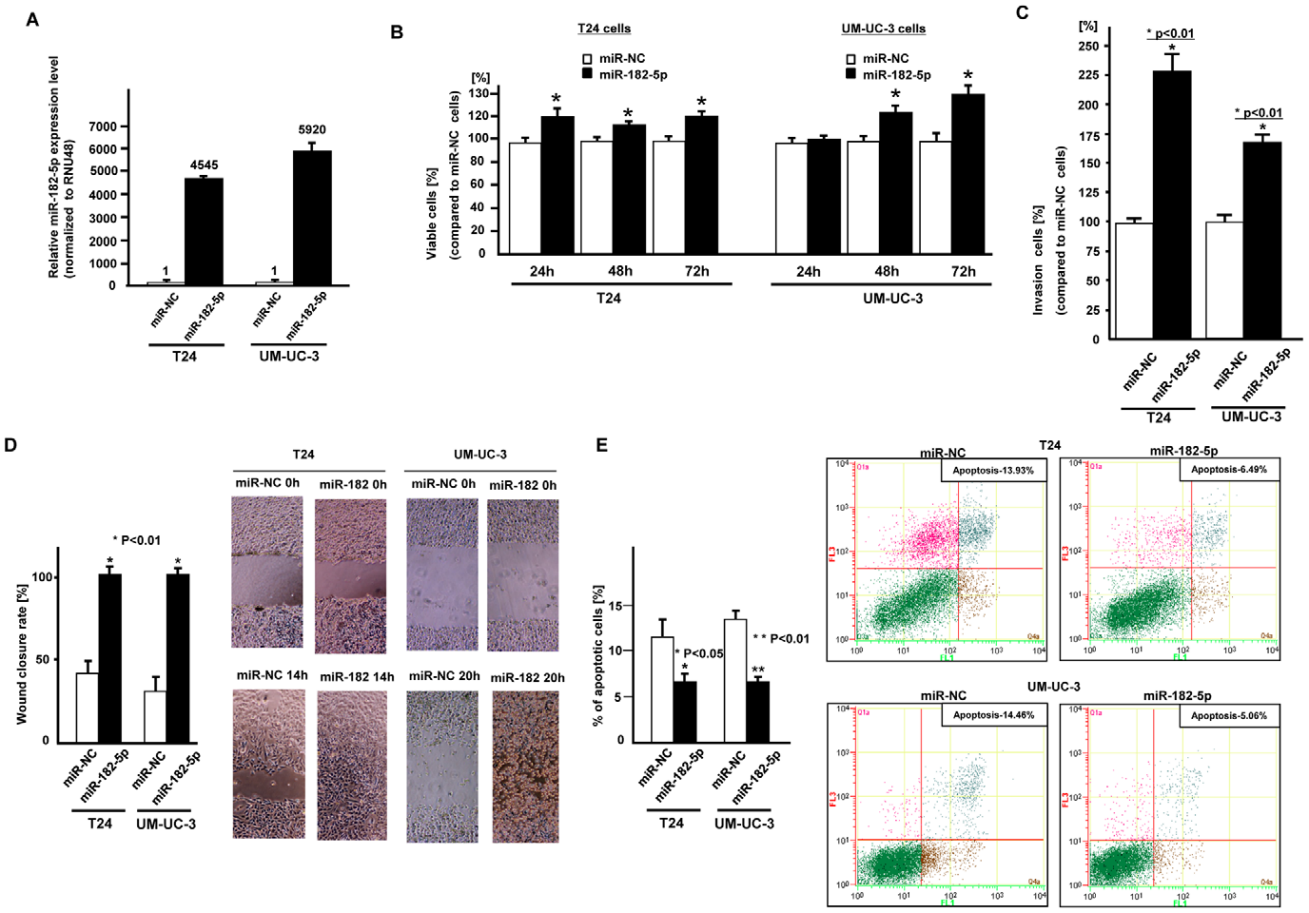
Effect of miR-182-5p over-expression on bladder cancer cell function (T24, UM-UC-3). Two bladder cancer cell lines (T24 and UM-UC-3) were transiently transfected with either miR-182-5p precursor or control (miR-NC). A. Relative miR-182-5p expression, B. Cell viability assay, C. Invasion assay, D. Wound healing assay (24 hours), E. Flow cytometric analysis of apoptosis in miR-NC or miR-182-5p transfected BC cells.

To confirm the function of miR-182-5p, we transfected miR-182-5p precursor into bladder cancer cell lines (T24 and UM-UC-3). At 24 hours after transfection of miR-NC or miR-182-5p precursor into bladder cancer cells, the miR-182-5p expression level was verified by real time PCR (fold change; 4545, 5920, respectively **Fig. 2** **-A**). Then several functional analyses were performed. We observed significantly increased cell proliferation (**Fig. 2** **-B**), invasion (**Fig. 2** **-C**) and migration (**Fig. 2** **-D**) in miRNA-182-5p transfected cells compared to miR-NC transfected cells. In addition, miR-182-5p significantly decreased cell apoptosis (**Fig. 2** **-E**).

### 3′-UTR-Luciferase Assay and Target Protein Expression in miR-182-5p Transfectants

**Figure 3:**
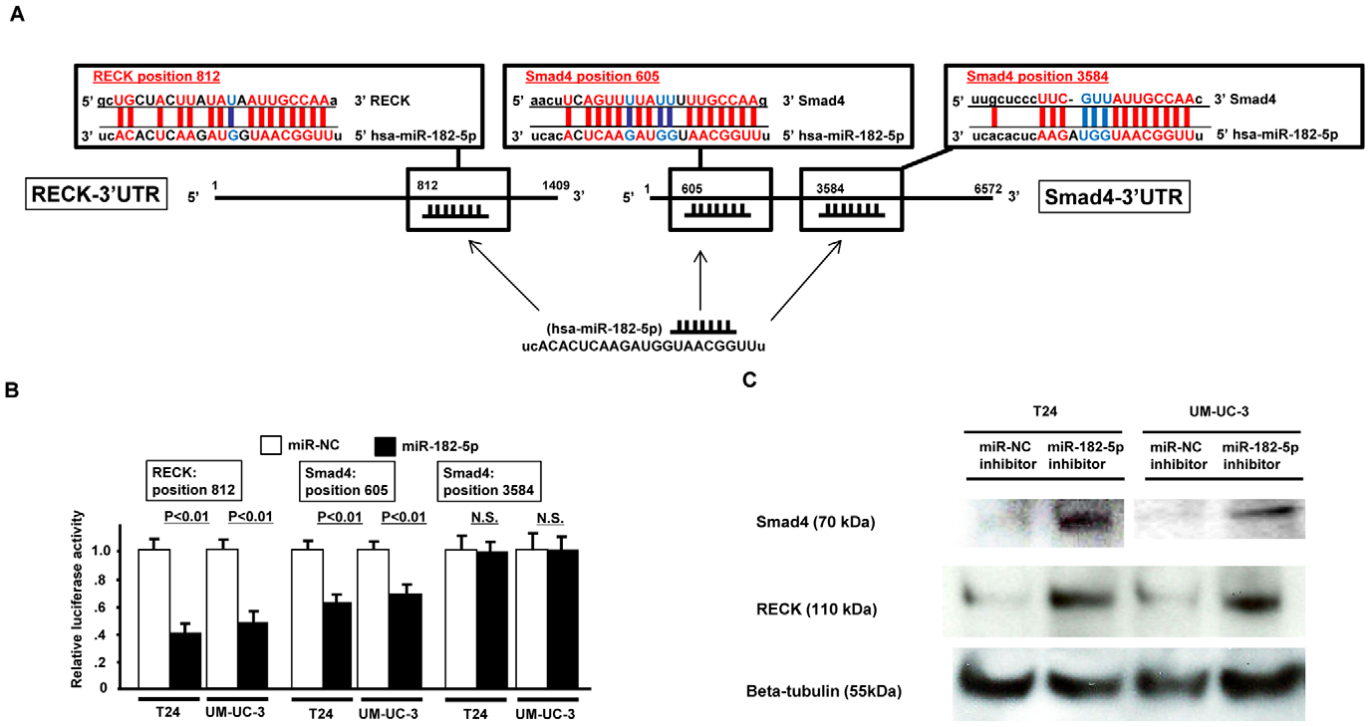
miR-182-5p binds to the 3′ UTR of RECK and Smad4 mRNAs and down-regulates expression. A. RECK and Smad4 3′UTR position and complementary miR-182-5p sequences. B. 3′UTR Luciferase assay (miR-NC and miR-182-5p precursor), C. RECK, Smad4 and beta-tubulin protein expression in miR-NC inhibitor or miR-182-5p inhibitor transfected bladder cancer cells (T24, UM-UC-3).

RECK mRNA has one while Smad4 has two potential complimentary binding site with miR-182-5p within its 3′ UTR (**Fig. 3** **-A**). Based on these results, we performed 3′UTR luciferase assays and found that the relative luciferase activities with these sites were significantly decreased in miR-182-5p transfected bladder cancer cells (T24, UM-UC-3) (**Fig. 3** **-B**). These results suggest that RECK and Smad4 mRNAs are potential target genes of miR-182-5p. Western analysis confirmed that RECK and Smad4 protein expression was significantly increased in miR-182-5p inhibitor transfected cells (**Fig. 3** **-C**).

### Effects of Over-expression of RECK and Smad4 on Bladder Cancer Cell (T24) Function

**Figure 4:**
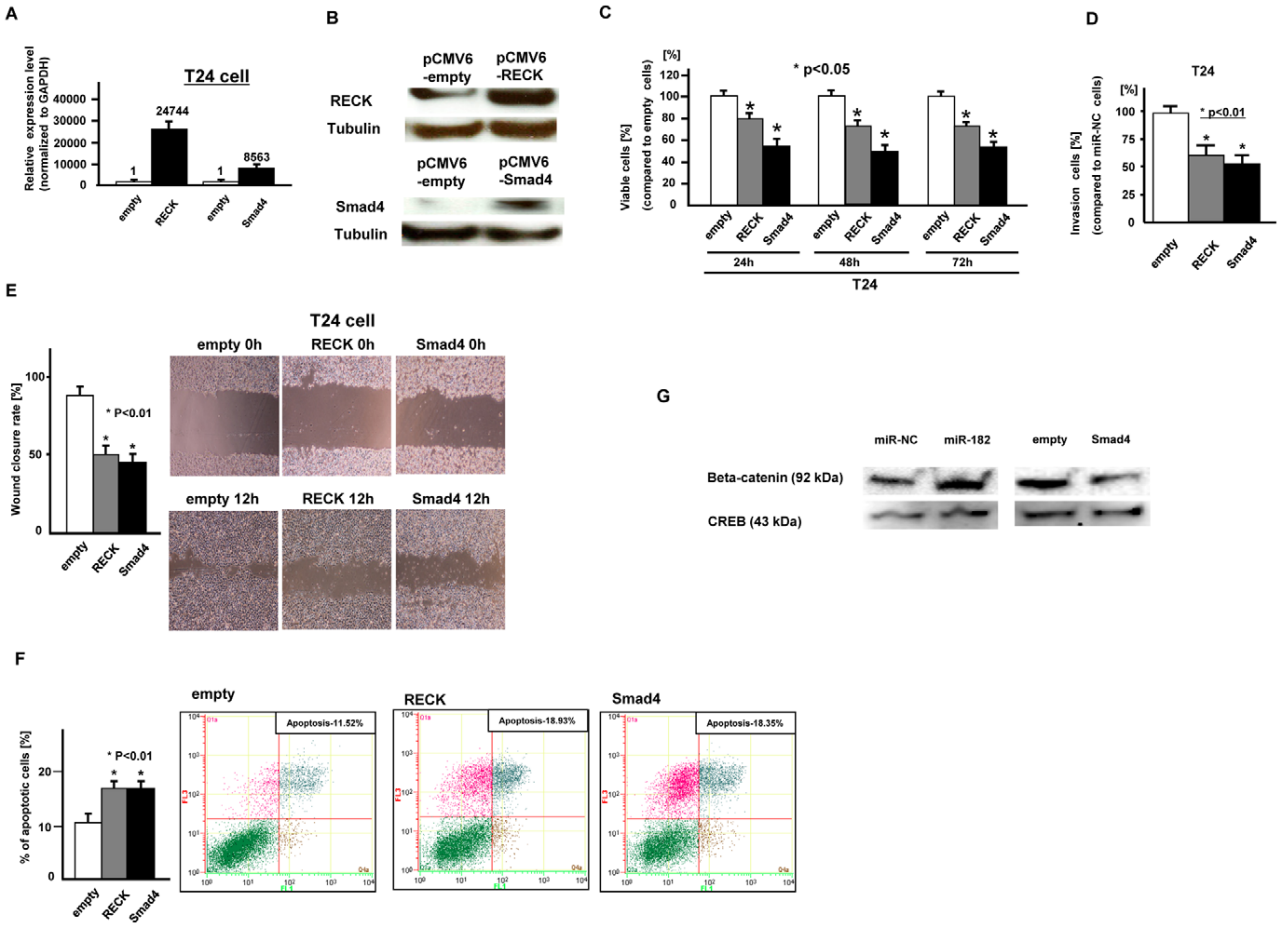
Effect of RECK and Smad4 over-expression on bladder cancer cell (T24) function. A. At 24 hours after transfection of either pCMV6-empty, pCMV6-RECK or pCMV6-Smad4 into bladder cancer cells (T24), RECK and Smad4 expression levels were verified by real time RT-PCR (fold change; 24744, 8563, respectively) and Western analysis (B) **C.** Cell viability assay, D. Invasion assay, E. Wound healing assay, F. Flow cytometric analysis of apoptosis in empty, RECK and Smad4 transfected T24 cells. Data are the mean ± S.D. of four independent experiments. G. beta-catenin expression in nuclear fraction, CREB was used as control.

To look at the function of RECK and Smad4, we overexpressed RECK and Smad4 in T24 cells which was confirmed by measuring mRNA (**Fig. 4** **-A**) and protein expression levels (**Fig. 4** **-B**). Then we performed several functional analyses. As shown in Figure 4, cell viability (**Fig. 4** **-C**), invasion (**Fig. 4** **-D**) and migration (**Fig. 4** **-E**) were significantly inhibited in RECK and Smad4 transfected T24 bladder cancer cells. As shown in **Figure 4** **-F**, the percentage of apoptotic cells was significantly increased in RECK or Smad4 transfected cells.

### Effect of miR-182-5p and Smad4 on Beta-catenin Expression in the Nuclear Fraction

As shown in **Figure 4** **-G**, beta-catenin expression in the nuclear fraction was significantly increased in miR-182-5p transfected T24 cells. In contrast, beta-catenin expression in the nuclear fraction was significantly decreased in Smad4 transfected T24 cells.

## Discussion

A number of microRNAs have been identified as tumor suppressor or oncogenes based on their expression level in bladder cancer tissues and/or functional analysis. However, many miRNA studies have focused on tumor suppressor miRNAs including miR-125b, -133a, -143, -145, -200 family (200a, 200b, 200c, 141, 429), -203, -205, -218, -449a, -493 and -517a [21]–[29]. Two miRNAs (miR-21 and miR-129) have been identified and confirmed as oncogenes in bladder cancer [30], [31].

MiR-182-5p has also been reported to be an oncogene in several cancers [32]–[37]. Similar to our results, one report found that miR-182-5p expression was significantly higher in bladder cancer tissues compared to normal urothelium, however functional analysis was not performed in this study [38]. Thus we investigated the relationship between miR-182-5p expression and clinical parameters including pathological stages and patient outcomes and found that miR-182-5p expression was correlated to shorter overall survival after operation in bladder cancer patients. These results suggest that miR-182-5p may be a new and useful diagnostic biomarker in bladder cancer.

Our next aim was to determine whether miR-182-5p functions as a bladder cancer oncogene. Of three bladder cancer cell lines (T24, UM-UC-3, J82), the expression of miR-182-5p in two cell lines (T24 and UM-UC-3) was in the range of expression observed in bladder cancer tissues. Thus we used these two bladder cancer cell lines (T24 and UM-UC-3) for functional analysis experiments. We found that over-expression of miR-182-5p significantly promoted bladder cancer cell viability, migration and invasion and inhibited apoptosis. We used several algorithms to search for potential miR-182-5p target genes since microRNAs exert their effects by regulating target gene expression. We identified RECK and Smad4 as potential target genes by 3′UTR luciferase assay and Western analyses.

MMP plays an important role in cancer invasion and metastasis and MMP-2 elevation in bladder cancer tissues has been reported to be correlated with tumor stage and poor prognosis in bladder cancer patients [39], [40]. RECK is a crucial MMP-2 repressor and RECK expression was previously reported to be down regulated in bladder cancer tissues compared to normal urothelium [16], [17]. In addition, DNA methylation has been identified as a RECK silencing mechanism [41]. Recently miR-21 was identified as a regulator of *RECK* gene expression in several cancers [42], but to date there has been no report showing direct regulation of RECK by miR-182-5p.

We also investigated the function of RECK by over expressing it in a bladder cancer cell line (T24). As shown, RECK inhibited cell proliferation, migration and invasion abilities in bladder cancer cells and the number of apoptotic cells was increased by RECK transfection.

Smad4 is an important signal transduction component of TGF-beta and recent studies show that Smad4 functions by cooperating with beta-catenin in several cancers.

Wnt-beta catenin signaling is crucial for embryogenesis and tumourigenesis [43]. In cancer cells, the Wnt pathway is usually activated causing unphosphorylated beta-catenin to accumulate in the cytoplasm and moves to the nucleus, where it binds to TCF/LEF and transcriptionally regulates Wnt target genes promoting tumorigenesis [43]. In bladder cancer, deregulated Wnt-beta-catenin signaling plays an important role in progression and metastasis. Thus we looked to see whether beta-catenin expression was altered by either miRNA-182 or Smad4 transfection. As we observed, miR-182-5p increased nuclear beta-catenin expression while Smad4 decreased nuclear beta-catenin expression. As far as we know, there have been no reports about miR-182 and Wnt-beta-catenin signaling and our results suggest that onco-miR-182-5p may be involved in the regulation of Wnt-beta-catenin related genes.

In our study, Smad4 overexpression decreased bladder cancer cell proliferation, migration and invasion ability. Apoptosis was also increased with Smad4 overexpression in bladder cancer cells. Since loss of Smad4 has been reported to play a causal role in initiating squamous cell carcinomas of the skin, upper digestive tract as well as adenocarcinoma of the gastrointestinal tract [44], our results may indicate that Smad4 plays an important role in bladder cancer. Since we focused only on Smad4 in the Wnt-signaling cascade, it is possible that other genes may be directly or indirectly regulated by miR-182-5p. Additional experiments will be needed to elucidate the exact role of miR-182-5p in Wnt-beta catenin signaling. Taken together, this study shows that miR-182-5p exerts its oncogenic effects in bladder cancer cells by down-regulating RECK and Smad4.

This is the first report to also document that miR-182-5p expression is significantly increased in bladder cancer tissues where it functions as an oncogene by inhibiting RECK and Smad4 expression and may be potentially useful as a prognostic biomarker. These results also suggest that miR-182-5p may have therapeutic potential for the treatment of bladder cancer.

## Materials and Methods

### Ethics Statement

Formalin-fixed, paraffin-embedded (FFPE) bladder cancer samples were obtained from the San Francisco Veterans Affairs (VA) Medical Center. Written informed consent was obtained from all patients and the study was approved by the UCSF Committee on Human Research (Approval number: H9058-35751-01).

### Clinical Samples

A total of 18 male patients with pathologically confirmed bladder cancer were enrolled in this study (Veterans Affairs Medical Center at San Francisco).

### Cell Culture

Three bladder cancer cell lines (J82; ATCC number: HTB-1, T24; ATCC number: HTB-4, UM-UC-3; ATCC number: CRL-1749) were purchased from the American Type Culture Collection (ATCC, Manassas, VA). The J82 and UM-UC-3 cell lines were cultured in MEM Eagle’s with Earle’s BSS supplemented with 10% fetal bovine serum. The T24 cell line was cultured in McCoy’s 5A medium with 10% fetal bovine serum.

### RNA and Protein Extraction

RNA (microRNA and total RNA) was extracted from formalin-fixed, paraffin-embedded (FFPE) human bladder cancer and non-cancerous normal bladder tissues (urothelial cells) using a miRNeasy FFPE kit (Qiagen) after laser micro-dissection based on pathologists reviews. Total RNA was also extracted from bladder cancer cell lines using a miRNeasy mini kit (QIAGEN). Cells were lysed with RIPA buffer (Thermo Scientific, Rockford, IL) containing protease inhibitors (Sigma, St. Louis, MO). Protein quantification was done using a BCA protein assay kit (Thermo Scientific, Rockford, IL). The NE-PER Nuclear and Cytoplasmic Extraction Reagent was used to extract nuclear and cytoplasmic protein fractions from bladder cancer cells (Thermo Fisher Scientific, Rockford, IL).

### MicroRNA Transfection (pre-miR Precursor and miR Inhibitor)

Pre-miR™ miRNA precursors [negative control (miR-NC) or hsa-miR-182-5p, Ambion] were transiently transfected into bladder cancer cells by Lipofectamine 2000 (Invitrogen) according to the manufacturer’s instructions.

Anti-miR™ miRNA inhibitor [negative control (inh-NC) or miR-182-5p inhibitor (miR-182 inhibitor), Ambion] were transiently transfected into bladder cancer cells by siPORT NeoFX Transfection Agent (Ambion) according to the manufacturer’s instructions. After transfection, cells were incubated at 37°C for 48 hours until assessment.

### Cell Viability, Cell Invasion, Wound Healing Assay

Cell viability was measured 3 days after transfection with MTS (CellTiter 96 Aqueous One Solution Cell Proliferation Assay, Promega). Data are the mean ± S.D. of 6 independent experiments. Cell invasion assays were performed with the CytoSelect 24-well cell invasion assay kit (Cell BioLab, San Diego, CA) according to the manufacturer’s instructions. Transfected cells were re-suspended in culture medium without FBS and placed in the upper chamber in triplicate. After 48 hours incubation at 37^o^ C (5% CO2), cells migrating through the membrane were stained. The results were expressed as invaded cells quantified at OD 560nm. The wound healing process begins with tissue matrix remodeling, migration, and eventual closing of the wound area. Therefore this assay is frequently used for assessment of cancer cell migration. Wound healing assay was performed with the CytoSelect 24-well wound healing assay kit according to the manufacturer’s instructions. To generate a wound field, transfected cells were cultured until they formed a monolayer around the insert. After removing the insert, a 0.9 mm open wound field was generated and cells were allowed to migrate from either side of the gap. Wound closure was monitored and the percent closure was measured. [Percent closure rate (%) = migrated cell surface area/total surface area x100)].

### Apoptosis Analyses

Cells (48 hours after transfection) were washed twice with 1xPBS and trypsinized. After inactivating trypsin in complete medium, the cells were re-suspended in ice-cold 1x binding buffer (70 µl). Annexin V-FITC solution (10 µl) and 7-AAD viability dye (20 µl) were added to 70 µl of the cell suspensions. After incubation for 15 minutes in the dark, 400 µl of ice-cold 1x binding buffer was added. The apoptotic distribution of the cells in each sample was then determined using a FACS (Cell Lab QUANTA SC, Beckman Coulter, Fullerton, CA). Data are the mean ± S.D. of four independent experiments.

### Plasmid Construction and 3′UTR-Luciferase Assay

We constructed individual plasmids for each binding site in the 3′UTR of mRNA from potential target genes based on microRNA.org information. Then we confirmed miR-182-5p binding to the target genes mRNA 3′UTR by luciferase assay with miR-182-5p precursor. PmirGLO Dual-Luciferase miRNA Target Expression Vector was used to perform 3′UTR luciferase assay (Promega, Madison, WI, USA). The primer sequences used for plasmid inserts are shown in **Table 1**. In a total volume of 20 µl, 5 µl each of 100 µM forward primer and reverse primer, 2 µl of 10x annealing buffer (100 mM Tris-HCl, pH 7.5, 1 M NaCl, 10 mM EDTA) and 8 µl water were added to a 200 µl PCR tube and incubated at 95°C for 5 minutes then placed at room temperature for 1 hr. The oligonucleotides were ligated into the *Pme*I- *Xba*I site of pmirGLO Dual-Luciferase miRNA Target Expression Vector. Colony direct PCR was performed for insert recognition using REDTaq (Sigma, St. Louis, MO, USA). The primers used for PCR were as follows: forward primer, 5′-cgtgctggaacacggtaaaa-3′; reverse primer, 5′-gcagccaactcagcttcctt-3′. PCR parameters for cycling were as follows: 94°C for 3 minutes, 30 cycles of PCR at 94°C for 30 seconds, 55°C for 30 seconds and 72°C for 30 seconds, 72°C for 10 minutes and 4°C for 10 minutes. The PCR product was digested with NotI (TaKaRa/Fisher Scientific, *Pittsburgh*, *PA, USA*). The sizes of vectors containing inserts were about 200 bp and 100 bp by electrophoresis since the NotI recognition sequence was incorporated into the primers. For miR-182-5p precursor transfection, bladder cancer cells were co-transfected with miR-NC and pmirGLO or miR-182-5p and pmirGLO Dual-Luciferase miRNA Target Expression Vectors using Lipofectamine 2000 (Invitrogen). Luciferase activity was assessed using the Dual-Luciferase® Reporter Assay System (Promega) (48 hours after their transfection).

**Table 1:** Primer sequences used for plasmid construction.

name		sequence	
**RECK NheI cloning forward primer**	**5'**	**GCTAGCggccaagctgggtccgagcatcccg**	**3'**
**RECK XhoI cloning reverse primer**	**5'**	**CTCGAGcaactacaaaccagcagtcctgaat**	**3'**
**Smad4 NheI cloning forward primer**	**5'**	**GCTAGCttgcttcagaaattggagacatatt**	**3'**
**Smad4 XhoI cloning reverse primer**	**5'**	**CTCGAGattttgtagtccaccatcctgataa**	**3'**
**RECK-S**	**5'**	**AAACTAGCGGCCGCTAGTgcTGCTACTTATATAATTGCCAAaT**	**3'**
**RECK-AS**	**5'**	**CTAGAtTTGGCAATTATATAAGTAGCAgcACTAGCGGCCGCTAGTTT**	**3'**
**Smad4-1S**	**5'**	**AAACTAGCGGCCGCTAGTaactTCAGTTTTATTTTTGCCAAgT**	**3'**
**Smad4-1AS**	**5'**	**CTAGAcTTGGCAAAAATAAAACTGAagttACTAGCGGCCGCTAGTTT**	**3'**
**Smad4-2S**	**5'**	**AAACTAGCGGCCGCTAGTttgctcccTTCGTTATTGCCAAcT**	**3'**
**Smad4-2AS**	**5'**	**CTAGAgTTGGCAATAACGAAgggagcaaACTAGCGGCCGCTAGTTT**	**3'**

### Overexpression Plasmid of Target Genes (RECK, Smad4) and Functional Analyses

In order to construct target gene (RECK, Smad4) over expressing plasmids, the genes were amplified with total RNA from human adult normal kidney tissues (catalog#: R1234142-50, Biochain Institute, Newark, CA) and RWPE-1 by transcription–polymerase chain reaction (RT-PCR). The sequences of primers for cloning are shown in **Table 1**. Polymerase chain reaction products were cloned into the pTargeT-Mammalian Expression Vector System (Promega, Madison, WI). Then pCMV6-RECK or pCMV6-Smad4 was obtained by subcloning a NheI–XhoI fragment from pTargeT-RECK/Smad4 into the NheI–XhoI site of pCMV6-Entry Vector.

Initially we transfected pCMV6-empty and pCMV6-RECK or -Smad4 into bladder cancer cells and RNA and protein were extracted. Overexpression of RECK or Smad4 was confirmed by real time RT-PCR and Western Blot analysis and functional analyses were performed.

### Quantitative Real-time RT-PCR

Quantitative real-time RT-PCR was performed in triplicate with an Applied Biosystems Prism 7500 Fast Sequence Detection System using TaqMan universal PCR master mix according to the manufacture’s protocol (Applied Biosystems Inc., Foster City, CA, USA). The TaqMan probes and primers were purchased from Applied Biosystems. Human GAPDH and RNU48 were used as an endogenous control. Levels of RNA expression were determined using the 7500 Fast System SDS software version 1.3.1 (Applied Biosystems).

### Western Analysis

Total cell protein (15–20 µg) was used for Western blotting. Samples were resolved in 4–20% Precise Protein Gels (Thermo Scientific, Rockford, IL) and transferred to PVDF membranes (Amersham Biosciences, Fairfield, CT). The membranes were immersed in 0.3% skim milk in TBS containing 0.1% Tween 20 for 1 hour and probed overnight at 4 4°C with primary polyclonal and monoclonal antibody against Smad4 (#9515), RECK (#3433), beta-catenin (#9562), CREB (#9197) and beta-tubulin (#2128) from Cell Signaling Technology, Beverly, MA. Blots were washed in TBS containing 0.1% Tween20 and labeled with horseradish peroxidase (HRP)-conjugated secondary anti-mouse or anti-rabbit antibody (Cell Signaling Technology, Beverly, MA). Proteins were enhanced by chemiluminescence (Amersham ECL plus Western Blotting detection system, Fairfield, CT) for visualization. The protein expression levels were expressed relative to beta-tubulin or CREB levels.

### Statistical Analysis

All statistical analyses were performed using StatView (version 5; SAS Institute Inc., NC). A *p*-value of <0.05 was regarded as statistically significant.
